# Levels of depressive and anxious symptoms of pregnant women before vs. during the COVID-19 pandemic

**DOI:** 10.1192/j.eurpsy.2021.1068

**Published:** 2021-08-13

**Authors:** M. Barros, M. Aguiar, A. Macedo, J. Azevedo, A.T. Pereira

**Affiliations:** 1 Departamento De Ciências Naturais, Universidade do Sudoeste da Bahia - UESB, Vitória da Conquista, Bahia, Brazil; 2 Institute Of Psychological Medicine, Faculty Of Medicine, University of Coimbra, Coimbra, Portugal; 3 Institute Of Psychological Medicine, Faculty Of Medicine, University of Coimbra, coimbra, Portugal

**Keywords:** anxiety and depression, pandemic, Fear, pregnancy

## Abstract

**Introduction:**

The effects on the population’s mental health due to the rapid global spread of COVID-19 are even greater for specific groups such as pregnant women.

**Objectives:**

To compare levels of depressive and anxiety symptoms of pregnant women before vs. during the COVID-19 pandemic and to analyze the role of COVID-19 fear in perinatal psychological disorder.

**Methods:**

200 Brazilian women evaluated during the pandemic in May-June 2020 (Sample-1) with the Brazilian Covid-19 Fear Scale for the Perinatal Period (Barros et al. 2020) and Screening for Perinatal Depression and the Perinatal Anxiety Crawl Scale, both with α> .90. Sample-1 was compared with a sample of 300 Portuguese women; these responded to the same questionnaires, before the pandemic, in 2017 and 2018 (Sample-2).

**Results:**

Sample-1 had significantly higher mean scores of depression (52.73 ± 20.26 vs. 35.87 ± 16.98, t = 10.77, p <.001) and anxiety (36.58 ± 18.23 vs. 18.50 ± 13.71, t = 11.94, p <.001) and correlated significantly (p <.05) and moderate (r.30) with the fear of COVID-19. Hierarchical regression analyzes showed that, even after controlling for the effect of risk factors for PPP (Pereira et al. 2020), fear of COVID-19 is a significant predictor of depressive symptomatology levels (increments of 2-5%) and anxious (10-15%) during the pandemic.

**Conclusions:**

The Sample-1 being from a different country may be a confusing factor, however, the magnitude of differences in PPP levels and the relevant role of fear in COVID-19, alert us to be aware of perinatal mental health.

